# expowo: An R package for mining global plant diversity and distribution data

**DOI:** 10.1002/aps3.11609

**Published:** 2024-07-30

**Authors:** Débora C. Zuanny, Bruno Vilela, Peter W. Moonlight, Tiina E. Särkinen, Domingos Cardoso

**Affiliations:** ^1^ Instituto de Biologia Universidade Federal da Bahia, Rua Barão de Jeremoabo s.n., Ondina Salvador 40170‐115 Bahia Brazil; ^2^ School of Botany Trinity College Dublin 1 College Green, Dublin 2 Ireland; ^3^ Royal Botanic Garden Edinburgh, 20a Inverleith Row Edinburgh EH3 5LR Scotland United Kingdom; ^4^ Instituto de Pesquisas Jardim Botânico do Rio de Janeiro (JBRJ) Rua Pacheco Leão 915, 22460‐030, Rio de Janeiro Rio de Janeiro Brazil

**Keywords:** biodiversity, data mining, natural history collections, vascular plants

## Abstract

**Premise:**

Data on plant distribution and diversity from natural history collections and taxonomic databases are increasingly becoming available online as exemplified by the Royal Botanic Gardens, Kew's Plants of the World Online (POWO) database. This growing accumulation of biodiversity information requires an advance in bioinformatic tools for accessing and processing the massive data for use in downstream science. We present herein expowo, an open‐source package that facilitates extracting and using botanical data from POWO.

**Methods and Results:**

The expowo package is implemented in R and designed to handle the entire vascular plant tree of life. It includes functions to readily distill taxonomic and distributional information about all families, genera, or species of vascular plants. It outputs a complete list of species in each genus of any plant family, with the associated original publication, synonyms, and distribution, and plots global maps of species richness at the country and botanical country levels, as well as graphs displaying species‐discovery accumulation curves and nomenclatural changes over time. To demonstrate expowo's strengths in producing easy‐to‐handle outputs, we also show empirical examples from a set of biodiverse countries and representative species‐rich and ecologically important angiosperm families.

**Conclusions:**

By harnessing bioinformatic tools that accommodate varying levels of R programming proficiency, expowo functions assist users who have limited R programming expertise in efficiently distilling specific botanical information from online sources and producing maps and graphics for the further interpretation of biogeographic and taxonomic patterns.

Botanical collections provide extensive information to support science, education, and conservation efforts (Graham et al., 2004; Paton et al., 2020; Walters and Pence, 2021; Westwood et al., 2021). Open and publicly accessible botanical data sets have greatly improved our ability to produce comprehensive floristic checklists, taxonomic revisions, and phenology estimates, particularly for poorly studied tropical floras (Funk, 2003; Bebber et al., 2010; Cardoso et al., 2017; Besnard et al., 2018; Sweeney et al., 2018; Park et al., 2023). To that end, a future goal is to create a “global metaherbarium” by digitizing biodiversity data across the global botanical network of herbaria, which could become a key source of information on plant diversity and distribution (Davis et al., 2022). However, attaining this goal also requires developing bioinformatic solutions that facilitate interactive investigation and easy download of these extensive botanical data sets. This ensures the accessibility and manipulation of data for different users and downstream scientific fields, including taxonomy, conservation, ecology, evolution, and biogeography.

Large biodiversity databases have been assembled to support worldwide scientific research and conservation efforts at any geographic or taxonomic level. Current best examples of such databases from which extensive botanical information can be accessed include (i) the Global Biodiversity Information Facility (GBIF; https://www.gbif.org), which holds billions of specimen records from museums and herbaria worldwide; (ii) the International Plant Names Index (IPNI; https://www.ipni.org), which provides a comprehensive list of all published angiosperm names; (iii) the GenBank platform of the National Center for Biotechnology Information (NCBI; https://www.ncbi.nlm.nih.gov), which holds publicly accessible DNA sequence data; and (iv) the more recently assembled global taxonomic databases of all currently accepted plant species and their distribution, such as the World Flora Online (WFO; https://www.worldfloraonline.org) and the World Checklist of Vascular Plants (WCVP; Govaerts et al., 2021) available through the Royal Botanic Gardens, Kew's Plants of the World Online (POWO; https://powo.science.kew.org), which are nomenclaturally and taxonomically curated and continuously updated by experts. Except for the taxonomically verified databases like WFO and WCVP, most of these databases are supplied with information from the digitization processes of primary biodiversity data (Krishtalka and Humphrey, 2000; Soberón and Peterson, 2004), and not all of this information is readily available for use (Peterson et al., 2018). Additionally, mistakes during data entry, translation between databases, and typographical errors can lead to misleading distribution and diversity patterns, causing severe inaccuracies in species lists (Maldonado et al., 2015; Cardoso et al., 2017). More recently, the issue of data standardization has become increasingly important, especially because multiple databases are often required to address complex research questions (Willemse et al., 2008).

Computational tools with capabilities to enable data access, processing, cleaning, and analysis are needed in order to tackle common issues involving biological information and to increase the value of botanical collections. For example, the use of GBIF data for understanding plant distribution and diversity patterns without expert checks, synonymy cleaning, and removal of records of cultivated occurrences has been identified as problematic in several studies (Goodwin et al., 2015; Cardoso et al., 2017). To more efficiently standardize the biodiversity data available, some recently created R packages have been developed to work with selected databases as taxonomic references. For example, the lcvplants package is grounded on the Leipzig Catalog of Vascular Plants (LCVP; Freiberg et al., 2020), the WorldFlora package enables plant name matching against the WFO taxonomic backbone data (Kindt, 2020), and the flora package (Carvalho, 2022) interacts with the Flora e Funga do Brasil taxonomic database (http://floradobrasil.jbrj.gov.br). Other packages, such as the rgbif (Chamberlain et al., 2024), CoordinateCleaner (Zizka et al., 2019), and bdc (Ribeiro et al., 2022), are extraordinarily useful tools for accessing and cleaning available plant biodiversity records via GBIF (e.g., Maldonado et al., 2015; Guedes et al., 2018; Moonlight et al., 2020). Their combined use with expertly validated taxonomic and distribution data from global databases, such as WFO and WCVP, ensures a more reliable and significant step in data cleaning. For example, a recent study investigating the global diversification of orchids employed such a combined approach (Pérez‐Escobar et al., 2024) to remove as many misidentified records as possible, which may impact downstream analyses (Maldonado et al., 2015). Their approach simply involved filtering the original record database sourced from GBIF by excluding entries where the distribution did not align with the species distribution per botanical countries as provided by the WCVP‐curated geographical distribution data.

Here, we introduce the new package expowo implemented in R (R Core Team, 2024) to perform automated searches to extract plant taxonomic and geographical records of the expert‐validated WCVP database. Unlike other R packages that deal with large botanical data sets by using application programming interface (API) or secure file transfer protocol (SFTP) servers, such as rWCVP (Brown et al., 2023; https://CRAN.R-project.org/package=rWCVP), WorldFlora (Kindt, 2020; https://cran.r-project.org/web/packages/WorldFlora), and flora (Carvalho, 2022; https://CRAN.R-project.org/package=flora), expowo was developed to interact with POWO by parsing the HTML source code embedded in each displayed webpage of a plant taxon. Although rWCVP was also designed to mine the WCVP database (Brown et al., 2023), it retrieves distribution information limited to the Biodiversity Information Standards (TDWG) geographical codes (Brummitt, 2001)—here indicated as botanical country–level subdivision—and does not have user‐friendly functions to easily explore the global distribution of species richness and the dynamics of species nomenclatural changes over time. To enhance user‐friendliness, the functions within our package are designed to cater to users without advanced programming expertise. These functions require only minimal input arguments to process data that might otherwise demand numerous lines of code using different packages. We showcase the potential of expowo using two examples, from which we produce a list of all accepted plant species for a set of biodiverse countries, lists of all accepted species for a set of economically and ecologically important families with associated distribution maps, and graphics displaying species accumulation and nomenclatural changes in selected genera of grasses.

## METHODS AND RESULTS

### Package description

The expowo R package is an open‐source tool comprising eight major functions, five auxiliary functions, and three associated data packages. It is specifically designed for extracting taxonomic and distribution data of species and genera for any family (or a list of families) of vascular plants from the WCVP database that is publicly available in the POWO taxonomic database. The package was written in R (3.5.0 or later) under the MIT license, and the latest version is available on CRAN (https://CRAN.R-project.org/package=expowo; see Data Availability Statement); the developmental version is available on GitHub (https://github.com/DBOSlab/expowo/).

The package workflow includes selecting the most appropriate function for the desired data, defining how the package will handle the input data, and then checking, extracting, and saving the data. Taxonomic results may be returned in a dataframe‐formatted object or saved as a comma‐separated values (CSV) file in a newly created folder within the designated working directory. The results include the number of accepted species for any genus, as well as the full list of accepted species in any genus or family, their authorship, original publication, and global distribution at the country and botanical country levels. The figure‐plotting functions displaying global maps of species richness and graphics with taxonomic information over time were built using ggplot2 (Wickham, 2009), which is founded on the grammar of graphics (Wilkinson, 2005). Leveraging the versatile features of ggplot2, the expowo‐derived plots based on taxonomic and distribution data can be fully customized by freely modifying or adding any layer.

The expowo functions provide user‐friendly accessibility for extracting, saving, and plotting taxonomic and distribution data, making it approachable for individuals with no prior programming knowledge. This deliberate design includes distinct yet interconnected and relatively similar functions tailored for users without coding expertise to obtain specific taxonomic and distribution results more efficiently. This versatility makes the package suitable for introductory courses to the R language, with practical examples applied in botany and biodiversity, facilitating an effective learning experience. To further aid usage and comprehension, we also offer complete tutorials for each function, with examples and detailed vignettes, on the package website (https://dboslab.github.io/expowo/).

### Package content

**Figure 1:**
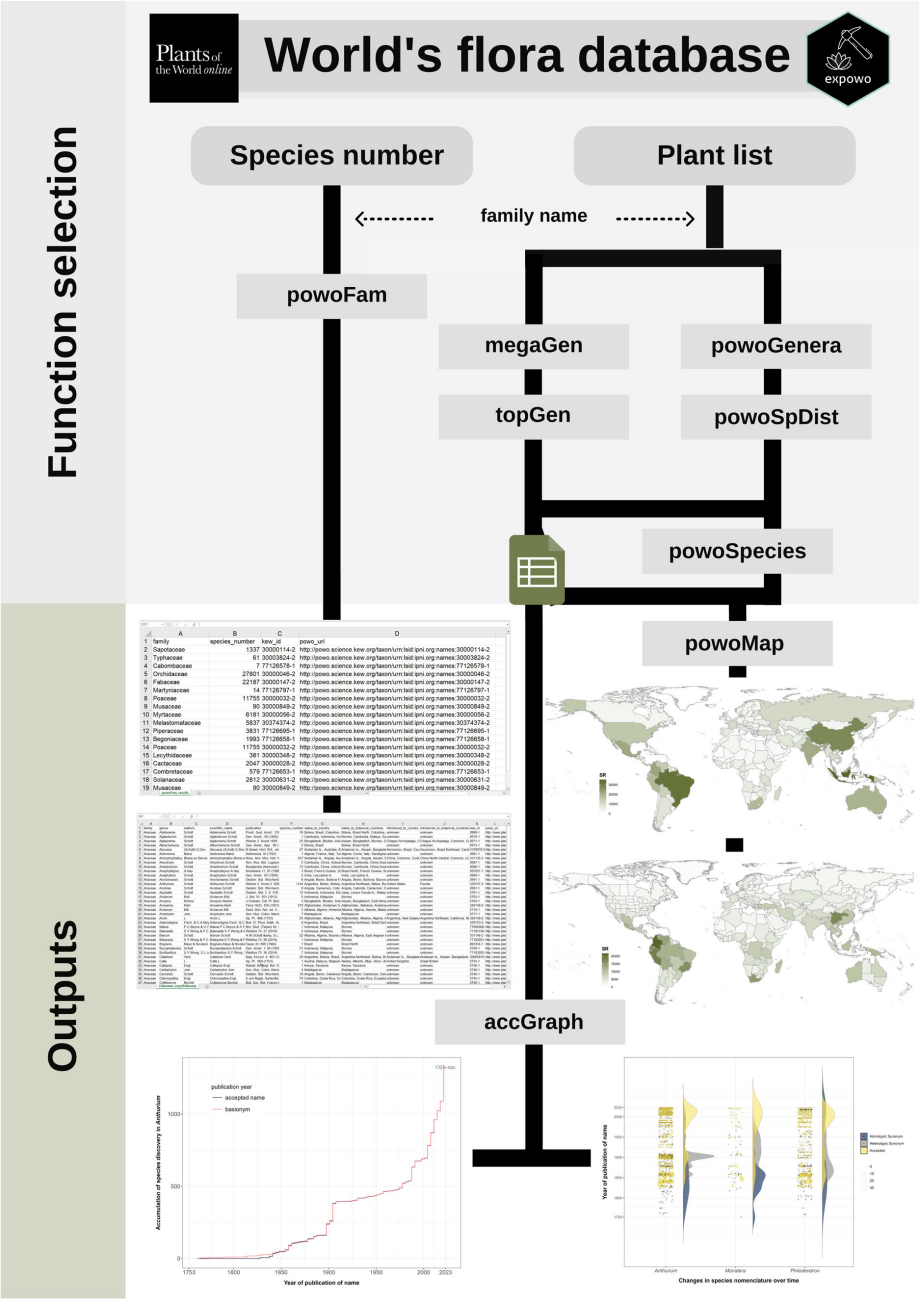
Graphical representation of the structure and major functions of expowo for mining the World Checklist of Vascular Plants (WCVP) database through Kew's Plants of the World Online (POWO) website. The package is structured to achieve two main objectives for plant data extraction: providing the species number for any genus or family and a complete species list with associated global distribution. The arrows with dashed lines represent the mandatory input information required for running the functions. The solid black lines connect the functions and their possible outputs: simple spreadsheets (top) and complete spreadsheets (bottom), global distribution maps of species richness according to political country division (top) and botanical countries (bottom), and graphics of cumulative number of species names (left) and nomenclatural changes over time (right).

The package includes six main functions for extracting taxonomic and distribution data (*powoFam*, *powoSpDist*, *powoGenera*, *powoSpecies*, *megaGen*, *topGen*; Table 1), in addition to two main functions for plotting maps and graphics (*powoMap*, *accGraph*; Table 1). The mining functions work with other main auxiliary functions (e.g., *getInfo* and *saveCSV*) and require only the name of the target family or a vector with multiple family names (Figure 1). To streamline searches that may halt unexpectedly for any reason, the functions were designed to support resuming a paused search and recommencing from the most recently retrieved taxon. Together, these functions can mine selected data and provide full spreadsheets, global distribution maps of species richness, and graphics displaying species‐discovery accumulation curves and nomenclatural changes through time (Figure 1).

**Table 1:** All functions included in the expowo package and associated descriptions related to their specific functionalities when interacting with Kew's Plants of the World Online (POWO).

Function	Description
*megaGen*	Extract megadiverse genera from POWO
*topGen*	Extract topmost species‐rich genera of any plant family from POWO
*powoFam*	Extract species number of any plant family from POWO
*powoGenera*	Extract list of genera of any family from POWO
*powoSpecies*	Extract list of species from POWO
*powoSpDist*	Extract list of species distributions from POWO
*powoMap*	Create global maps of species richness
*accGraph*	Create graphics of accumulation of species discoveries and changes in species nomenclature over time

### Diversity data extraction

Table‐formatted lists can be extracted from R by inputting the name of a plant family (or a vector of plant family names). Each major function automatically establishes a connection with the associated POWO webpage by internally calling the respective uniform resource identifier (URI) of the queried family from the data package POWOcodes. General examples on how to use expowo functions are also provided in the package documentation. Because expowo uses the HTML source code embedded in each taxon page at POWO to mine the data, an internet connection is required.

The function *powoFam* was designed to count the total number of species within any family and to save the queried results in a spreadsheet. The *megaGen* function uses a specified threshold number to return a list of all megadiverse genera for any family of vascular plants and their associated number of accepted species. The default value of the threshold in the *megaGen* function is based on Frodin (2004), who reviewed large plant genera with more than 500 species. The function *topGen* is similar to *megaGen*; however, while *megaGen* selects all genera with species above a specified number, *topGen* filters the topmost species‐rich genera within any family, which does not necessarily mean those genera are megadiverse. The function *powoGenera* generates a genus‐level list with associated authorship, original publication of the protologue, the number of species in each genus, the Kew ID, and webpage URI, while the *powoSpecies* function generates a species‐level list with information about the associated authorship, original publication, the native distribution according to country and botanical country, the introduced distribution according to country and botanical country, the Kew ID, and the webpage URI. The function *powoSpDist* generates the same type of spreadsheet, but it is built to focus specifically on a designated list of species, streamlining the process of retrieving species distribution information. In contrast, *powoSpecies* is designed to retrieve all species from a designated list of families and/or genera. Both the *powoSpecies* and *powoSpDist* functions will automatically assign as unknown any species missing information about the native or introduced distribution. Despite yielding similar outputs, the utility of *powoSpecies* and *powoSpDist* depends on the user's initial interest. If the interest is centered on a particular species, *powoSpDist* offers a more efficient approach, eliminating the need to run a code that mines data for an entire family or genus and subsequently filter the desired species—a process that could be more time‐consuming. This targeted approach allows for a more streamlined and resource‐efficient search.

### Distribution data extraction

The original POWO distribution information is limited to the TDWG's botanical country–level subdivision (level 3), without detail provided on political country–level distribution. Although some of TDWG's botanical country–level distributions coincide with political boundaries of countries, this is not always the case. This may be somewhat problematic for comparative analyses in country‐level conservation studies, because most effective conservation programs are often designed by national‐level initiatives (Martins et al., 2017; Ren et al., 2019). To overcome this major distributional caveat within POWO, we created the associated data package botregions, which provides a dataframe with the global political country names and associated classification of botanical countries. By using this dataframe, all of expowo's functions that produce biodiversity lists can easily convert the botanical country distributions, as originally retrieved from POWO, into distributions across political country delimitation. Thus, the user does not need to write new lines of R code to perform such conversions because any list delivered by expowo will always include distributions at both the country and botanical country levels.

### Plotting species richness distribution

Our newly developed *powoMap* function uses the Natural Earth map data (https://www.naturalearthdata.com) framework to plot global‐scale maps of species richness at the country and botanical country levels. Despite being originally designed to produce maps for any specific taxonomic level extracted with expowo's *powoSpecies* function, where the level is defined by the argument ‘taxclas’, *powoMap* also accepts any dataframe‐formatted input data with a minimum requirement of one column with the binomial species name and the column(s) with corresponding distribution in the countries and/or botanical countries. A single map can be automatically produced for the entire input data, or multiple richness maps can be produced for individual taxonomic groups, in a single run by just specifying a column name with the associated taxonomic classification. Additionally, the *powoMap* function can produce global maps according to botanical countries by adjusting the arguments ‘botctrs’ and ‘distcol’. To generate elegant publication‐quality figures with fewer keystrokes that best represent the species richness data graphically, we have designed the *powoMap* function using specific functions from ggplot2 (Wickham, 2009). expowo also offers the option to display the color scales of the maps based on color palettes from both the Viridis (Garnier et al., 2024) and RColorBrewer R packages (Neuwirth, 2022), which provide visualizations that take into account the needs of users with color vision deficiency.

### Plotting species‐discovery accumulation and nomenclatural changes through time

The ggplot2‐based function *accGraph* was designed to investigate new species descriptions and nomenclatural synonymizations over time. To do this, the function produces plots depicting species‐discovery accumulation curves and violin plots of nomenclatural changes. It can automatically plot figures for any above‐genus taxonomic level or for an input data set focused on a geographic region. For example, if one is interested in visualizing the dynamics of nomenclatural changes of all flowering plants in a particular country, the *accGraph* function can be used to (1) mine the species list, constraining the distribution to that country; (2) add a new column in the resulting dataframe containing the same character (e.g., the name of the country) inside all rows; and (3) define this modified dataframe as input for the *accGraph* function, where the name of the newly added column is specified in the argument ‘spp_changes_col’.

### Comparison with the rWCVP package

Just like the recently launched package rWCVP (Brown et al., 2023), expowo accesses plant name and distribution data from the WCVP database. However, while rWCVP uses Kew's SFTP server to locally download a snapshot of the WCVP database, expowo mines the data directly from the POWO website. Unlike other packages that only work with a specific biodiversity database, expowo's functions for plotting global maps of species richness and graphs of species‐discovery accumulation and nomenclatural changes were built to be as general as possible, allowing them to work with input data sourced from any database, as long as the taxonomic and distribution data are properly formatted.

Because expowo was originally designed to extract data from the POWO website interface, its scope extends beyond the taxonomic and distributional data provided by rWCVP, enabling greater scalability for the retrieval of different types of information. This is because POWO hosts comprehensive taxonomic details, including morphological descriptions, uses, lifeforms, and other pertinent supporting bibliographic data. As such, in a forthcoming version of expowo, we envision enriching the package with intuitive and accessible functions to mine more general information for species across all plant families, particularly focusing on plant uses. This enhancement aims to bridge the gap between botanical science and traditional uses, facilitating a deeper understanding of plant diversity and its applications.

To assess the performance of expowo, we conducted a speed comparison with rWCVP for the task of extracting and plotting a distribution map for the genus *Psidium*. The tests were performed on the same computer running Windows 11, equipped with an AMD Ryzen 5 5500U 2.10‐GHz processor and 16 GB RAM. The trial with rWCVP took 19.56 seconds to extract and generate a map for the genus *Psidium*, whereas it took 3.52 minutes using expowo. If the task is only to plot a map, it finishes after 14.69 seconds using rWCVP, and after 2.58 seconds using expowo. While the total processing time was faster with rWCVP, it is important to note that this measurement does not account for the time required to download the data package rWCVPdata, which is a prerequisite for executing functions within rWCVP. Our package retrieves real‐time data from POWO, necessitating access to each taxon page via the respective URI. The speed of internet connectivity also plays a role during the data mining step as a potentially limiting factor. Although our package was faster in plotting a global map for *Psidium*, a Myrtaceae genus with 95 species, rWCVP may outperform expowo if the task involves extracting a much larger data set. It should also be noted that the expowo functions are configured to automatically insert a pause of 300 seconds after every 500th request by default. This is to prevent overloading the POWO server with too many requests in a short timeframe, which could potentially cause operational issues.

### Empirical examples

We demonstrate expowo's functionality with two sets of empirical examples: (1) extracting species lists at the country level for a set of biodiverse countries, and (2) extracting species lists at the family level for a set of economically and ecologically important plant families and mapping their diversity and distribution. See Appendix S1 for the scripts used to generate both the spreadsheets and the maps with the full search results.

#### Extracting species lists at the country level

**Figure 2:**
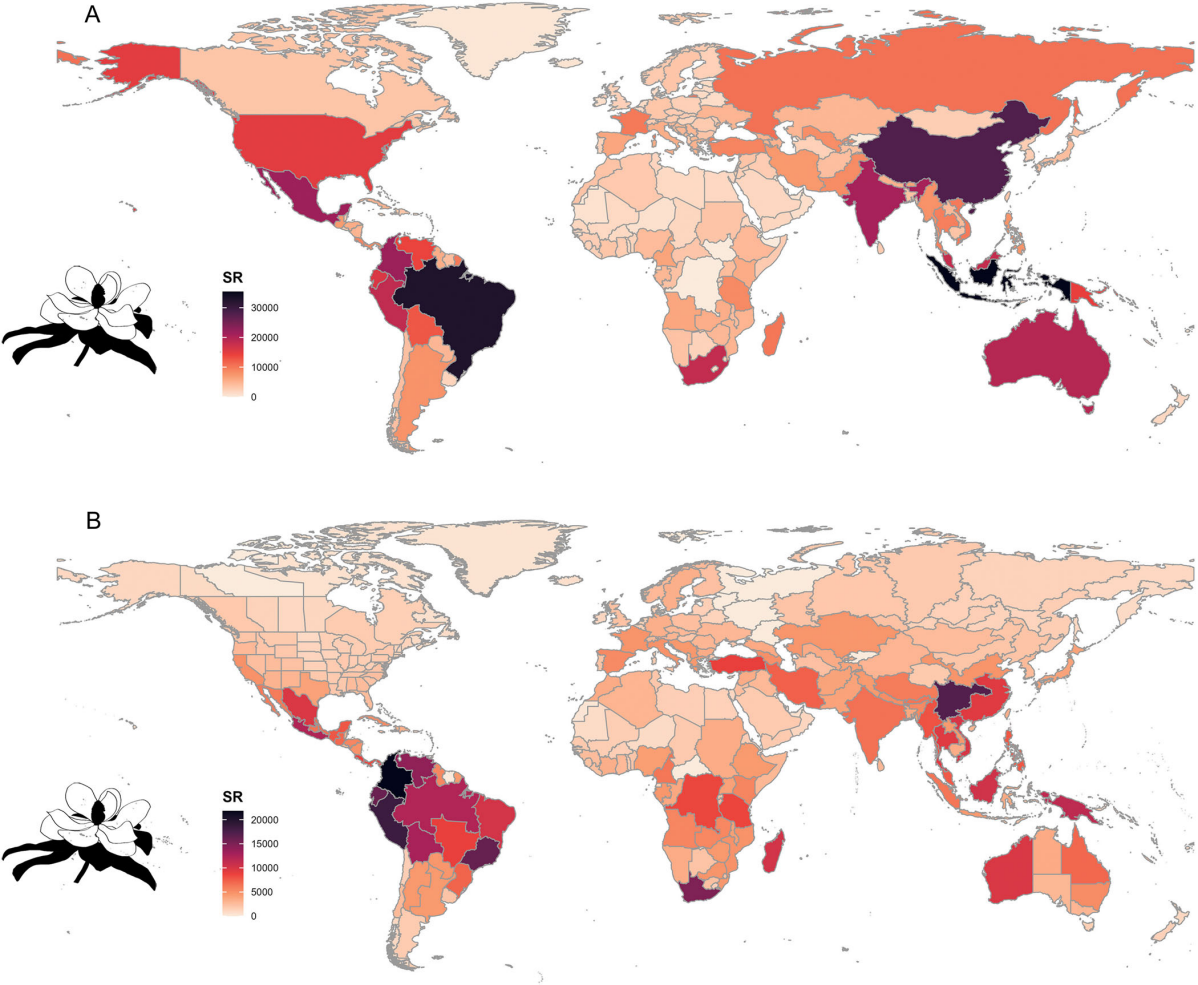
The global native distribution of all currently accepted angiosperm species mapped according to (A) political countries and (B) botanical countries. The data were extracted from Kew's POWO using expowo's *powoSpecies* function and plotted using the *powoMap* function. The color scale representing the amount of species richness is based on the “rocket” color palette from the Viridis package (Garnier et al., 2024). The silhouettes of *Magnolia grandiflora* are from PhyloPic (http://phylopic.org/).

We first used the *powoSpecies* function to extract a list of accepted species of all angiosperm families available at POWO and generated global maps of species richness according to two different divisions of the world (Figure 2). These maps show Brazil and Indonesia to be the most speciose countries at the country level, whereas Colombia is the most species rich at the botanical country level. We then selected some countries that are renowned for their high levels of species richness and endemism to produce their POWO‐based list of accepted species. While several tropical countries are among the world's most biodiverse, efforts to summarize their plant biodiversity have been made for only a few of them (e.g., Brazil, The Brazil Flora Group, 2022; Papua New Guinea, Cámara‐Leret et al., 2020; Mexico, Ulloa‐Ulloa et al., 2017; Madagascar, Antonelli et al., 2022). Our results showed the following: Brazil (32,864 spp., Appendix S2), Papua New Guinea (12,306 spp., Appendix S3), Mexico (21,539 spp., Appendix S4), Malaysia (14,672 spp., Appendix S5), Madagascar (10,263 spp., Appendix S6), Indonesia (27,700 spp., Appendix S7), and Thailand (9481 spp., Appendix S8). These species lists can serve as a starting point for directing actions in compiling a country checklist for those megadiverse countries where comprehensive documentation of their flora is still lacking. Additionally, they may serve to compare with already established country‐level online flora databases such as Flora e Funga do Brasil (The Brazil Flora Group, 2022) in order to know whether concerted efforts by taxonomists working regionally are also reflected in synergistic updates in global databases.

#### Extracting family‐level species lists and generating visualizations of species richness and historical nomenclature changes

Three economically and ecologically important plant families (Poaceae, Fabaceae, and Begoniaceae) were used as examples for how to use the expowo package to extract global lists of accepted species and genera, as well as to produce maps of species richness. We first queried the POWO database with the function *powoSpecies* in October (for Begoniaceae data) and November 2022 (for Poaceae and Fabaceae data). POWO currently accepts 11,811 species and 790 genera in Poaceae (Appendix S9), 22,186 species and 778 genera in Fabaceae (Appendix S10), and 1994 species and two genera in Begoniaceae (Appendix S11). In contrast, in the WFO Plant List (https://wfoplantlist.org/plant-list), there are 12,648 species of Poaceae, 23,942 species of Fabaceae, and 2196 species of Begoniaceae. While these figures show diverging species numbers across databases, we believe it is not related to the accuracy of the underlying data, as all of them are taxonomically validated. Rather, these differences are likely due to the frequency at which the databases are continuously maintained and updated.

**Figure 3:**
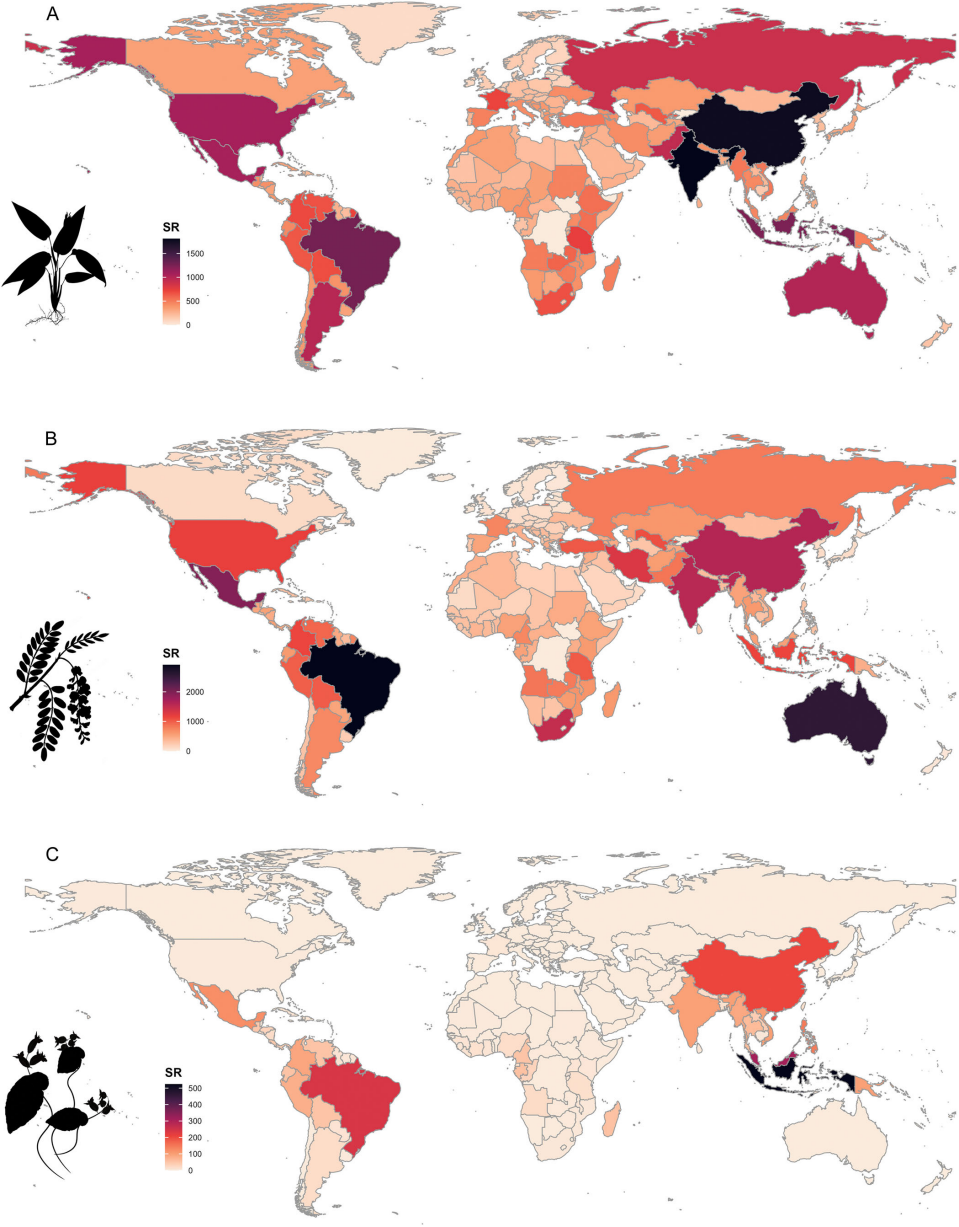
The global native distribution of all accepted angiosperm species mapped according to the political country division of the world within (A) Poaceae, (B) Fabaceae, and (C) Begoniaceae. The data were extracted from Kew's POWO using expowo's *powoSpecies* function and plotted using the *powoMap* function. The color scale representing the amount of species richness is based on the “rocket” color palette from the Viridis package (Garnier et al., 2024). The silhouettes of *Anomochloa marantoidea*, *Robinia pseudoacacia*, and *Begonia* sp. (from PhyloPic, http://phylopic.org/) present the typical morphology of each species as representatives of their respective families.

**Figure 4:**
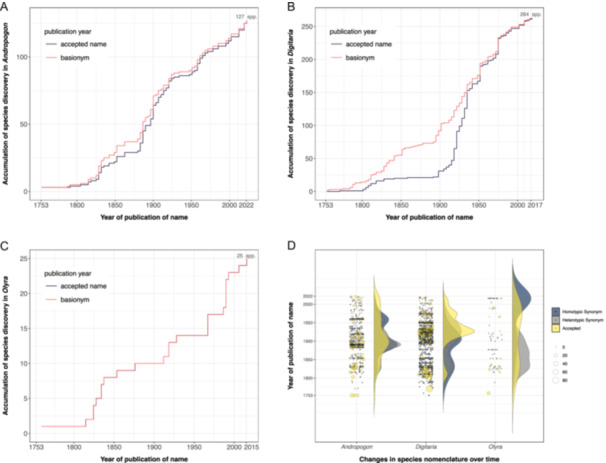
The dynamics of species description (A–C) and historical nomenclatural changes (D) of selected genera within Poaceae, as generated with expowo's function *accGraph*. The disparities in the curves depicting species description over time (A, B) arise from distinct approaches to account for actual discoveries. The black line is based on the publication dates of currently accepted species names, while the red line reflects the publication year of the basionym of each currently accepted species name. Thus, they illustrate the accumulation of formally described species, disregarding subsequent purely nomenclatural changes. In (D), the dynamics of nomenclatural changes is visually represented through varying sizes of curves and circles spanning from 1753 to 2022. Currently accepted names (yellow) are sized proportionately to their associated homotypic or heterotypic synonyms (shades of gray) published over time. Note that *Digitaria*, for example, has two peaks of relatively old accepted species (in yellow) with greater numbers of associated synonyms (in different shades of gray): a smaller yellow peak between 1800 and 1825, and a larger peak between 1900 and 1950.

Another strength of expowo compared to similar packages is its user‐friendly graphical representation of global species richness, particularly the taxonomically informative graphics depicting historical nomenclature changes. The extracted data can be represented graphically with global maps of species richness at country level for those plant families (Figure 3). The maps show that China and India have the most species for Poaceae, while Brazil, Australia, and Tasmania are the most species‐rich countries for Fabaceae, and Indonesia is the most species‐rich country for Begoniaceae. We also selected three genera within Poaceae (*Andropogon*, *Digitaria*, and *Olyra*) to show the other possible outputs generated by expowo: the creation of accumulation curves of species discoveries and nomenclatural changes over time (Figure 4). Each graphic displayed different patterns, highlighting a peak of synonymizations of *Andropogon* around 1890, while for *Digitaria* and *Olyra* synonymizations occurred over a longer time period.

## CONCLUSIONS

Here, we have presented the new R package expowo by detailing its functionality for taxonomic and geographic data extraction of the authoritative and taxonomically verified POWO database on global plant distribution and diversity. We further direct the package's users to utilize the vignettes provided on the website, which contains tutorials with examples. The expowo package represents another important step in facilitating and complementing the use of biological databases to foster further downstream botanical research. Specifically, the continued improvement of the package can increase scientific reproducibility and facilitate the identification of types of data that need standardization and refined curation, while generating publication‐ready spreadsheets and figures. By simplifying the extraction of plant data and the mapping of their spatial distribution, the package represents an important advance in documenting the world's flora that is accessible for users with limited R programming expertise. As data become increasingly accessible, it will be possible for researchers and conservationists to take into account the distribution of endangered species and make better decisions about conservation strategies and the delimitation of priority areas for funding. The ability of the expowo package to produce native and introduced distribution areas for species, genera, and families is also of potential interest for a multitude of research questions on biogeography and ecology.

## AUTHOR CONTRIBUTIONS

D.C.Z. and D.C. conceived the study and led the writing of the manuscript. D.C.Z. and D.C. wrote all R functions, the package, and the associated website. D.C. provided expertise on taxonomic aspects of the package, B.V. technically revised some R codes, D.C.Z. gathered the data and drafted the manuscript, and D.C.Z., T.E.S., and P.W.M. designed the empirical examples. All authors revised the text and approved the final version of the manuscript.

## Supporting information


**Appendix S1.** Scripts using expowo functions to replicate the examples for: (i) extracting and plotting the data from all species of angiosperms, (ii) extracting and plotting the data for each selected plant family, and (iii) extracting and plotting the data for the grass genera *Andropogon*, *Olyra*, and *Digitaria*.

**Appendix S2.** List of all POWO's accepted species of angiosperms with native distribution occurrence in Brazil, as retrieved with expowo's function *powoSpecies*.

**Appendix S3.** List of all POWO's accepted species of angiosperms with native distribution occurrence in Papua New Guinea, as retrieved with expowo's function *powoSpecies*.

**Appendix S4.** List of all POWO's accepted species of angiosperms with native distribution occurrence in Mexico, as retrieved with expowo's function *powoSpecies*.

**Appendix S5.** List of all POWO's accepted species of angiosperms with native distribution occurrence in Malaysia, as retrieved with expowo's function *powoSpecies*.

**Appendix S6.** List of all POWO's accepted species of angiosperms with native distribution occurrence in Madagascar, as retrieved with expowo's function *powoSpecies*.

**Appendix S7.** List of all POWO's accepted species of angiosperms with native distribution occurrence in Indonesia, as retrieved with expowo's function *powoSpecies*.

**Appendix S8.** List of all POWO's accepted species of angiosperms with native distribution occurrence in Thailand, as retrieved with expowo's function *powoSpecies*.

**Appendix S9.** List of all POWO's accepted species of Poaceae, as retrieved with expowo's function *powoSpecies* and used for mapping the global native distribution with *powoMap* to generate Figure 3A.

**Appendix S10.** List of all POWO's accepted species of Fabaceae, as retrieved with expowo's function *powoSpecies* and used for mapping the global native distribution with *powoMap* to generate Figure 3B.

**Appendix S11.** List of all POWO's accepted species of Begoniaceae, as retrieved with expowo's function *powoSpecies* and used for mapping the global native distribution with *powoMap* to generate Figure 3C.

## Data Availability

The expowo package is available on CRAN (stable version, https://CRAN.R‐project.org/package=expowo) and on GitHub (developmental version, https://github.com/DBOSlab/expowo). Usage instructions and tutorials are available on the package website (https://dboslab.github.io/expowo/). All data used in this paper are presented in the Supporting Information.
