# Supplementary material for: expowo: An R package for mining global plant diversity and distribution data
Source: Appl Plant Sci. 2024 Jul 30;12(6):e11609. doi: 10.1002/aps3.11609 (PMC11610411; doi:10.1002/aps3.11609)
Supplement: Supplementary file 1 — Appendix S1. Scripts using expowo functions to replicate the examples for: (i) extracting and plotting the data from all species of angiosperms, (ii) extracting and plotting the data for each selected plant family, and (iii) extracting and plotting the data for the grass genera Andropogon, Olyra, and Digitaria. [file APS3-12-e11609-s001.pdf]

DOI 10.1002/aps3.11609

Appendix S1. Scripts using expowo functions to replicate the examples for: (i) extracting and plotting the data from all species of angiosperms, (ii) extracting and plotting the data for each selected plant family, and (iii) extracting and plotting the data for *Andropogon*, *Olyra*, and *Digitaria*.

Article title: expowo: An R package for mining global plant diversity and distribution data

Authors: Débora C. Zuanny, Bruno Vilela, Peter W. Moonlight, Tiina E. Särkinen and Domingos Cardoso

Contents

Reproducing the examples

Usage 1

Extracting and plotting the data from all species of angiosperms 2

Extracting and plotting the data for each selected plant family 3

Extracting and plotting the data for *Andropogon*, *Olyra*, and *Digitaria* 5

Usage

We have created **expowo** to automatize the process of extracting data from Kew's Plants of The World Online (POWO) database using R. Our goal is to pull species lists with all associated data of any vascular plant family. The empirical examples presented in the paper make use of this new R package. If you do not already have it, install **expowo** from GitHub and load it with:

```
install.packages("devtools")
devtools::install_github("DBOSlab/expowo")
library(expowo)
```

Here we will show how to extract data of all plant species and for each selected plant family in the paper, and then use this data to plot global maps of species richness distribution.

DOI 10.1002/aps3.11609

Extracting and plotting the data from all species of angiosperms

To create the dataset with all angiosperm species available at POWO, use the following script:

```
utils::data("POWOcodes")

angio <- subset(POWOcodes, (group %in% c("angiosperm")))

all_angio <- expowo::powoSpecies(family = angio$family,
                                 hybrid = FALSE,
                                 synonyms = TRUE,
                                 country = NULL,
                                 verbose = TRUE,
                                 rerun = FALSE,
                                 save = TRUE,
                                 dir = "results_powoSpecies",
                                 filename = "all_angio_species")
```

With this dataset, it was possible to filter for the species occurrence in selected species-rich countries. To generate figures of the global native distribution at the country and botanical country levels, use the newly created object "all_angio" as input in `powoMap`. Both global maps generated in this step were included in Figure 2 of the present paper.

```
# To create one map according to political countries
expowo::powoMap(inputdf = all_angio,
                botctrs = FALSE,
                distcol = "native_to_country",
                taxclas = NULL,
                verbose = FALSE,
                save = TRUE,
                vir_color = "rocket",
                bre_color = NULL,
                leg_title = "SR",
                dpi = 600,
                dir = "results_powoMap",
                filename = "Angiosperms_global_richness_country_map",
                format = "jpg")

# To create one map according to botanical countries
expowo::powoMap(inputdf = all_angio,
                botctrs = TRUE,
                distcol = "native_to_botanical_countries",
                taxclas = NULL,
```

DOI 10.1002/aps3.11609

```
                verbose = FALSE,
                save = TRUE,
                vir_color = "rocket",
                bre_color = NULL,
                leg_title = "SR",
                dpi = 600,
                dir = "results_powoMap",
                filename = "Angiosperms_global_richness_botcountry_map",
                format = "jpg")
```

Extracting and plotting the data for each selected plant family

We can extract the data and generate individual lists with all POWO's accepted species within some of the most diverse and ecologically important families (Begoniaceae, Fabaceae, and Poaceae) using a custom R script with the package **expowo**.

```
# Query for Poaceae using powoSpecies
poa_data <- expowo::powoSpecies(family = "Poaceae",
                                hybridspp = FALSE,
                                country = NULL,
                                verbose = TRUE,
                                save = TRUE,
                                dir = "results_powoSpecies",
                                filename = "all_species_Poaceae")

# Query for Fabaceae using powoSpecies
fab_data <- expowo::powoSpecies(family = "Fabaceae",
                                hybridspp = FALSE,
                                country = NULL,
                                verbose = TRUE,
                                save = TRUE,
                                dir = "results_powoSpecies",
                                filename = "all_species_Fabaceae")

# Query for Begoniaceae using powoSpecies
beg_data <- expowo::powoSpecies(family = "Begoniaceae",
                                hybridspp = FALSE,
                                country = NULL,
                                verbose = TRUE,
                                save = TRUE,
                                dir = "results_powoSpecies",
                                filename = "all_species_Begoniaceae")
```

DOI 10.1002/aps3.11609

Then, the function creates species lists with all POWO's accepted species of the selected plant families Poaceae, Fabaceae, and Begoniaceae. This data can be used for mapping the global native distribution with *powoMap*. The output generated with the following R code composed the Figure 3 of the present paper.

```
# To create one map for Poaceae according to political countries
expowo::powoMap(inputdf = poa_data,
                botctrs = FALSE,
                distcol = "native_to_country",
                taxclas = "family",
                verbose = FALSE,
                save = TRUE,
                vir_color = "rocket",
                bre_color = NULL,
                leg_title = "SR",
                dpi = 600,
                dir = "results_powoMap",
                filename = "Poaceae_global_richness_country_map",
                format = "jpg")

# To create one map for Fabaceae according to political countries
expowo::powoMap(inputdf = fab_data,
                botctrs = FALSE,
                distcol = "native_to_country",
                taxclas = "family",
                verbose = FALSE,
                save = TRUE,
                vir_color = "rocket",
                bre_color = NULL,
                leg_title = "SR",
                dpi = 600,
                dir = "results_powoMap",
                filename = "Fabaceae_global_richness_country_map",
                format = "jpg")

# To create one map for Begoniaceae according to political countries
expowo::powoMap(inputdf = beg_data,
                botctrs = FALSE,
                distcol = "native_to_country",
                taxclas = "family",
                verbose = FALSE,
                save = TRUE,
                vir_color = "rocket",
                bre_color = NULL,
                leg_title = "SR",
```

DOI 10.1002/aps3.11609

```
                dpi = 600,
                dir = "results_powoMap",
                filename = "Begoniaceae_global_richness_country_map",
                format = "jpg")
```

Extracting and plotting the data for *Andropogon*, *Olyra*, and *Digitaria*

We used three Poaceae genera to plot graphics showing accumulation of species discovery and historical nomenclatural changes. The four plots were combined and saved into Figure 4 using the functions *plot_grid* and *save_plot* from the **cowplot** package.

```
library(cowplot)

newdata <- powoSpecies(family = "Poaceae",
                       genus = c("Andropogon", "Olyra", "Digitaria"),
                       synonyms = TRUE,
                       save = FALSE)

accum_plots <- accGraph(inputdf = newdata,
                        verbose = TRUE,
                        spp_acc = TRUE,
                        spp_changes = FALSE,
                        spp_changes_col = NULL,
                        genus_plots = TRUE,
                        save = FALSE)

viol_plot <- accGraph(inputdf = newdata,
                      verbose = TRUE,
                      spp_acc = FALSE,
                      spp_changes = TRUE,
                      spp_changes_col = "genus",
                      genus_plots = FALSE,
                      save = FALSE)

all_plots <- cowplot::plot_grid(accum_plots[[1]],
                                accum_plots[[2]],
                                accum_plots[[3]],
                                viol_plot,
                                labels = c("(a)", "(b)", "(c)", "(d)"),
                                label_size = 25,
                                ncol = 2, nrow = 2,
                                align = "hv")
```

DOI 10.1002/aps3.11609

```
cowplot::save_plot("all_graphics_Poaceae.pdf",
                   all_plots,
                   ncol = 2,
                   nrow = 2,
                   base_height = 7.0,
                   base_aspect_ratio = 1.3,
                   base_width = 10)
```
